# Social isolation and loneliness among older adults in the context of COVID-19: a global challenge

**DOI:** 10.1186/s41256-020-00154-3

**Published:** 2020-06-05

**Authors:** Bei Wu

**Affiliations:** NYU Aging Incubator and Hartford Institute for Geriatric Nursing, 433 First Ave, 5th Floor, New York, NY 10010 USA

**Keywords:** COVID-19, Social isolation, Aging, Public health messaging

## Abstract

We are experiencing a historical moment with an unprecedented challenge of the COVID-19 global pandemic. The outbreak of COVID-19 will have a long-term and profound impact on older adults’ health and well-being. Social isolation and loneliness are likely to be one of the most affected health outcomes. Social isolation and loneliness are major risk factors that have been linked with poor physical and mental health status. This paper discusses several approaches that may address the issues of social isolation and loneliness. These approaches include promoting social connection as public health messaging, mobilizing the resources from family members, community-based networks and resources, developing innovative technology-based interventions to improve social connections, and engaging the health care system to begin the process of developing methods to identify social isolation and loneliness in health care settings.

## Background

We are experiencing a historical moment with an unprecedented challenge of the COVID-19 global pandemic. As of April 21st, 89 countries with more than a third of the global population have experienced lockdowns due to the outbreak. Many of the businesses that are currently closed are in some of the most prosperous cities like New York, Tokyo, and Paris. The coronavirus has swept through many parts of the world at a devastating and deadly speed, with over 200,000 people worldwide having died in countries struggling with shortages of healthcare workers, increasing sick people, and lack of personal protective equipment. Based on the death certificates of data retrieved and coded by the CDC National Center for Health Statistics, 78% of COVID-19 related deaths in the U.S. occurred among older adults age 65 and over [1]. Older adults are the segment of the population most vulnerable in this pandemic, largely due to their weaker immune systems and higher likelihood of having a chronic condition such as heart disease, diabetes, lung disease and cancer. Having any of these conditions is a risk factor for suffering complications from COVID-19. Globally, nursing home residents are affected the most. They have a much higher rate of death, and are more likely to be infected. In the UK., it is estimated that close to half of COVID-19 related deaths are now happening in care homes; in the U.S., about one-fifth of deaths occur in nursing homes [1]. In almost every country battling with the COVID-19 outbreak, older adults are being told to self-quarantine and shut themselves off from other people who might risk infecting them. In long-term care facilities, one common practice is to ban visits from family members and friends to these facilities in order to minimize the risk of spreading the virus. While these restrictions are legitimate under this time of crisis, they could have a significant negative impact on older adults’ mental health status, such as experiencing social isolation and loneliness.

## Social isolation and loneliness in the context of COVID-19

Social isolation is defined as the objective state of having few social relationships or infrequent social contact with others while loneliness is a subjective feeling of being isolated. Social isolation and loneliness are serious yet underestimated public health risks that affect a significant portion of the older adult population. In the U.S., approximately one-quarter of community-dwelling older adults are considered to be socially isolated, and 43% of them report feeling lonely [2]. The COVID-19 pandemic is increasing the number of older adults who are socially isolated including both community-dwelling older adults and nursing home residents, as many countries have issued stay-at-home orders and banned visits for nursing home residents.

Prior to the disease outbreak, the vast major of community-dwelling older adults actively participated in social activities, such as attending senior centers, churches activities, traveling, and many other social events. Community-based long-term care services are commonly available in developed countries and some developing countries. While family members can be primary caregivers caring for older adults with functional and cognitive impairment, community-based long-term care also plays an important role for many frail older adults. These community services and programs cover adult-day care, respite care, homemakers, meals on wheels, and home health services. For frail older adults living alone, meal delivery staff may be the only person they meet on a daily basis. For nursing home residents, family visits are an important way for them to feel socially connected, and family members are their link to the outside world. However, due to the lockdown policy, all these services and programs are no longer available. These restrictions would certainly increase social isolation and the feelings of loneliness of older adults. In the context of COVID-19, social isolation may be especially detrimental to family caregivers being that the majority are older adults themselves and are already at increased risk of stress, anxiety and depression [3].

Increasing evidence demonstrates that social isolation has detrimental impact on individual’s health and well-being. Studies, including our own [4], have found that social isolation and loneliness are major risk factors that have been linked with poor physical and mental health status: increased blood pressure, heart disease, obesity, diminished immune system functioning, depression, anxiety, poorer cognitive functioning, increased risk of Alzheimer’s disease, and mortality [4, 5]. Social isolation has been associated with an approximately 50% increased risk of developing dementia, a 29% increased risk of incident coronary heart disease and a 32% increased risk of stroke [2]. We need to be cognizant that the social isolation resulting from efforts to decrease the spread of COVID-19, can at the same time increase the risk of these negative outcomes, potentially having a profound impact on their health and wellbeing.

## Multiple approaches to decrease social isolation

### Public health messaging: maintaining social connection

While each country has asked their citizens to keep social distancing, this message of social distancing can be misleading. In fact, the public messaging of keeping physical distancing and maintaining socially connected is becoming more important than ever. Studies have shown that social support can mediate social isolation and improve mental health status [6, 7]. It is critical to mobilize the resources from family members, community-based networks and resources that address social isolation and loneliness in older adults. There is also great potential for older adults to be volunteers to provide much needed peer support for isolated individuals. In our early work, we found that many older adults, including the oldest old (age 85+), were actively providing support to their family, friends, and neighbors, such as providing companionship, giving comfort, cooking meals, and shopping for others [8]. In nursing homes, family and staff can play an essential role in helping residents socially connect through technology, such as video and social media.

### The role of technology in addressing social isolation

It is important to develop innovative technology-based interventions to improve social connection for this population. Mobile technologies can be instrumental, as they are transforming the way in which we interact with others, find information, access resources, and deliver services [9]. Our recent study found that 92% of the Chinese American older adults with low income and education had a smart device, and 72% used WeChat, the most commonly used social media software application in Chinese population. Anecdotal evidence and our personal observation suggest that Chinese Americans use social media applications such as WeChat to connect people from a distance to alleviate social isolation. It is time to develop more person-centered applications with the input from older adults and their family members. Existing evidence-based interventions for older adults can be used as the basis for creating needed social support via instant messaging apps or videos. In addition, peer support via social media may enhance the effects of evidence-based professional support, such as information resources, health promotion and counseling, and problem solving. On the other hand, we also need to be aware that as with other areas of health care, ethical and legal considerations especially must be explored when the technology is used in interventions for isolation and loneliness.

### Healthcare system’s response to social isolation

Globally, older adults have much higher usage of the health care system compared to younger populations. The health care system is well positioned to develop methods to identify social isolation and loneliness in health care settings. By identifying those at highest risk and whether their condition is acute or chronic, healthcare providers may be able to use these findings to develop appropriate clinical and public health interventions for patients. These interventions can then be adopted and implemented to other high-need countries/regions and populations served. Health professions schools and direct care worker training programs should include education and training related to social isolation and loneliness in their teaching curricula [2]. Development of more tele-health approaches can provide older adults and their family with better access to healthcare providers and facilitate screening, diagnosis, and treatment of social isolation.

## Conclusions

The outbreak of COVID-19 will have a long-term and profound impact on older adults’ health and well-being globally. Social isolation and loneliness are likely to become major risk factors that affect older adults’ health outcomes. Some strategies to address these issues can be implemented in many countries. These strategies include: raising awareness of the health and medical impact of social isolation and loneliness across the health care workforce and among members of the public; developing innovative technology based interventions to mobilize the resources from family members, community-based networks and resources that address social isolation and loneliness in older adults; and engaging the health care system to begin the process of developing methods to identify social isolation and loneliness in health care settings.

## Data Availability

Data sharing not applicable to this article as no data-sets were generated or analyzed during the current study.

## References

[1] National Centers for Health Statistics (2020). Provisional death counts for Coronavirus disease (COVID-19).

[2] National Academics of Sciences Engineering and Medicine (2020). Social isolation and loneliness in older adults: opportunities for the health care system.

[3] Wu B, Petrovsky DV, Wang J (2019). Dementia caregiver interventions in Chinese people: a systematic review. J Adv Nurs.

[4] DiNapoli EA, Wu B, Scogin F (2014). Social isolation and cognitive function in Appalachian older adults. Res Aging.

[5] Nicholson NR (2012). A review of social isolation: an important but underassessed condition in older adults. J Prim Prev.

[6] Gong X, Ni Z, Wu B (2020). The mediating roles of functional limitations and social support on the relationship between vision impairment and depressive symptoms in older adults. Ageing Soc.

[7] Wang J, Mann F, Lloyd-Evans B, Ma R, Johnson S (2018). Associations between loneliness and perceived social support and outcomes of mental health problems: a systematic review. BMC Psychiatry.

[8] Wu B, Yue Y, Silverstein NM, Axelrod DT, Shou LL, Song PP (2005). Are contributory behaviors related to culture? Comparison of the oldest old in the United States and in China. Ageing Int.

[9] Ni Z, Liu C, Wu B, Yang Q, Douglas C, Shaw RJ (2018). An mHealth intervention to improve medication adherence among patients with coronary heart disease in China: development of an intervention. Int J Nurs Sci.

